# A Multifactor Analysis of Fungal and Bacterial Community Structure in the Root Microbiome of Mature *Populus deltoides* Trees

**DOI:** 10.1371/journal.pone.0076382

**Published:** 2013-10-16

**Authors:** Migun Shakya, Neil Gottel, Hector Castro, Zamin K. Yang, Lee Gunter, Jessy Labbé, Wellington Muchero, Gregory Bonito, Rytas Vilgalys, Gerald Tuskan, Mircea Podar, Christopher W. Schadt

**Affiliations:** 1 Biosciences Division, Oak Ridge National Laboratory, Oak Ridge, Tennessee, United States of America; 2 Genome Science and Technology Program, University of Tennessee, Knoxville, Tennessee, United States of America; 3 Department of Microbiology, University of Tennessee, Knoxville, Tennessee, United States of America; 4 Department of Biology, Duke University, Durham, North Carolina, United States of America; Dowling College, United States of America

## Abstract

Bacterial and fungal communities associated with plant roots are central to the host health, survival and growth. However, a robust understanding of the root-microbiome and the factors that drive host associated microbial community structure have remained elusive, especially in mature perennial plants from natural settings. Here, we investigated relationships of bacterial and fungal communities in the rhizosphere and root endosphere of the riparian tree species *Populus deltoides*, and the influence of soil parameters, environmental properties (host phenotype and aboveground environmental settings), host plant genotype (Simple Sequence Repeat (SSR) markers), season (Spring vs. Fall) and geographic setting (at scales from regional watersheds to local riparian zones) on microbial community structure. Each of the trees sampled displayed unique aspects to its associated community structure with high numbers of Operational Taxonomic Units (OTUs) specific to an individual trees (bacteria >90%, fungi >60%). Over the diverse conditions surveyed only a small number of OTUs were common to all samples within rhizosphere (35 bacterial and 4 fungal) and endosphere (1 bacterial and 1 fungal) microbiomes. As expected, *Proteobacteria* and *Ascomycota* were dominant in root communities (>50%) while other higher-level phylogenetic groups (*Chytridiomycota, Acidobacteria*) displayed greatly reduced abundance in endosphere compared to the rhizosphere. Variance partitioning partially explained differences in microbiome composition between all sampled roots on the basis of seasonal and soil properties (4% to 23%). While most variation remains unattributed, we observed significant differences in the microbiota between watersheds (Tennessee vs. North Carolina) and seasons (Spring vs. Fall). SSR markers clearly delineated two host populations associated with the samples taken in TN vs. NC, but overall host genotypic distances did not have a significant effect on corresponding communities that could be separated from other measured effects.

## Introduction

Terrestrial plants experience complex interactions with microbes found immediately surrounding the root (rhizosphere) and inside of root tissues (endosphere). This is particularly true of perennial land plants where interannual climatic variability and extensive and long-lived root systems, that invade and occupy large volumes of soil, may increase the complexity of rhizospheric interactions. The microbiomes in these root-associated environments are comprised of bacteria, fungi, and to a lesser extent archaea, each with potential beneficial, neutral or detrimental effects on hosts' growth and development [1]–[6]. A thorough understanding of these complex relationships requires knowledge of resident microbes and factors shaping their abundance and community structure. Few studies have simultaneously examined bacterial and fungal root communities from the same host or genotype over time and even fewer have simultaneously and thoroughly measured the other associated physical, chemical, spatial and temporal factors that may affect these communities. Thus, a deeper analysis of root microbiome as a function of host and environmental factors is pivotal for expanding understanding of the nature and function of these relationships.

Native, woody perennial plant environments, such as those of cottonwood trees (*Populus* spp.), provide an ideal opportunity to understand these associations within relevant environmental settings. The importance of *Populus* spp. in the pulp and paper industry and their potential for future use in production of cellulose-derived biofuels, contributes incentive to increasing our understanding of the effects of microbial relationships on their growth and development. Additionally, *P. trichocarpa* was the first tree species to have a complete genome sequence [7] and several *Populus* species have become important plant model organisms for understanding the biology and ecology of woody perennials. Moreover, the possibility to study *Populus* in greenhouses, plantation agroecosytems, as well as in natural ecosystem settings where they can be dominant keystone species (especially in riparian zones) together make *Populus* spp. a powerful and relevant system for providing a better understanding of plant-microbe relationships.

The rhizosphere and endosphere microbiome of *Populus* is thought to be important to their overall health and development. *Populus*-associated bacteria are known to promote plant growth and development, increase disease resistance and improve phytoremediation potential [8]–[11]. Ectomycorrhizal (ECM) and arbuscular mycorrhizal (AM) relationships also are known to occur within *Populus* and influence plant growth and fitness [12], structure and composition of surrounding plants [13], and overall ecosystem functions [14]. Thus, characterizing the complex interactions between these trees and their microbiomes are an important step in understanding the overall properties of plants.

Several studies have focused on effects of either bacterial or fungal communities on *Populus* through sequencing cloned 16S rRNA genes and cultured representatives of the most abundant organisms [8]–[10]. We previously used high-throughput sequencing to characterize microbes associated with roots of *P. deltoides* and identified a clear distinction between communities in and on the roots (e.g., endosphere vs. rhizosphere) [15]. However, that study was limited to only a few individuals within two stands and did not address potential host or environmental factors that may structure microbial communities, or how these communities change over space and time. In other studies, mostly with agriculturally important plants and in greenhouse settings, host developmental stage, growing season, genotype/cultivar effects and soil properties have been shown to influence microbial community structure [16]–[22]. Deep-sequencing efforts that allow multiplexing of many samples simultaneously, such as the ones used in this study, present an opportunity to scale up these types of analyses and to potentially unravel the links between the *Populus* root microbiome and a wide variety of environmental and host factors that may shape them.

In this study two naturally occurring riparian populations of *Populus deltoides* occurring in Tennessee (TN) and North Carolina (NC) were investigated. We focused on examining the ecological and host factors that could lead to variation in the microbial diversity in and around natural root systems. Specifically, we correlated measures of root microbiome composition and structure with soil physical and chemical factors, host phenotypic factors and genotypic patterns (i.e., SSR-based genetic distances). We sampled roots over two seasons to discern the potential for seasonal variation within these communities. Finally, we described the distribution of OTUs among sampled trees and between rhizosphere and endosphere niches, and identified a core set of both fungal and bacterial OTUs in these two habitats that may play important roles within the plant-microbe-soil interface.

## Materials and Methods

### Study site and sampling

We collected native *P. deltoides* samples in two campaigns conducted in spring (May) and fall (September) of 2010. These samples were collected from multiple sites in two watersheds of North Carolina and Tennessee. A total of 23 trees were sampled each season, with eleven from NC and twelve from TN. The United States Army Corps of Engineers, the Tennessee Parks Department and various land owners generously provided access to sampling sites. All other sites were located within public right of way locations (roadsides, bridge crossings, boat launches, etc.) At each sampling site, we recorded the GPS coordinates with a handheld GPS unit. Three soil cores were taken from the adjacent area to each tree in spring sampling campaign only. These soils were refrigerated for further characterization performed at the Agricultural and Environmental Services Laboratory of the University of Georgia (http://aesl.ces.uga.edu/) on the sieved (4 mm) samples using the standard protocols available on their website. The characteristics of each tree and surrounding soil are presented in Tables S1 and S2.

We collected root samples by carefully excavating and tracing the roots back to the target *P. deltoides* as described in [15] to ensure identity of the individual roots sampled and correspondence between the host genotype and root samples. The collected root samples were stored on ice and processed the next day in lab. Tertiary fine roots were removed and loosely adhered soils were removed by shaking and washing with 100 ml of 10 mM NaCl solution to remove the adhering rhizosphere soil. The resultant wash was collected in 50 mL tubes, which was then defined as the rhizosphere sample. For endosphere samples, the surface of the same root samples were sterilized by rinsing first root sample an additional 4 times with sterile distilled water. The roots with diameter 2 mm or less were then transferred to 50 ml centrifuge tubes and washed using 6.15% of NaOCl with 2 to 3 drops of Tween 20 per 100 ml for 3 min, 100% ethanol for 30 s, and again with 3% of H_2_O_2_ for 30 s. These surface sterilized roots were then rewashed 3 additional times with sterilized distilled water. The sterility of the root surface was assessed by plating a subsample of surface disinfected root onto LB plates and incubating the plate overnight at 30°C. If contamination was found the procedure above was repeated. These surface sterilized root samples constitute endophyte samples.

### Detection of host microsatellite polymorphisms

Twenty microsatellites that previously showed clear polymorphisms in *P. trichocarpa* [23] and tested *P. deltoides* clones, were pre-selected for use in this study from a set of over 200. The PCR and SSR analytic protocols were as follows: reaction mixtures contained 25 ng of DNA, 50 ng of each SSR primer, 0.2 mM dNTPs, 0.5 U Taq DNA polymerase (Promega Corp., Madison, WI), 10 mM Tris-HCl (pH 8.3), 50 mM KCl, 2.0 mM MgCl2, 0.01% gelatin and 0.1 mg/ml bovine serum albumin. Amplification conditions on a GeneAMP 9700 thermocycler (Applied Biosystems, Foster City, CA) included an initial denaturation step at 94°C for 45 s followed by 30 cycles of 94°C for 15 s, 50–55°C for 15 s and 72°C for 1 min and concluded with a 5-min extension at 72°C. Reaction products were diluted up to 1∶200, denatured in HiDi formamide containing a 400-bp ROX standard (Applied Biosystems Foster City, CA) and processed on the ABI Prism 3730 DNA analyzer. GeneScan version 3.5 was used for size calling of raw alleles based on the internal standard and Genotype version 3.5 was used to visualize and assign alleles to categories for scoring purposes [23].

### Microbial DNA extraction and 454 pyrosequencing

For rhizosphere samples, 2.0 ml of rhizosphere material were pelleted via centrifugation. The resultant pellet was then used for extractions using a PowerSoil DNA extraction kit (MoBio, Carlsbad, CA). For endophyte samples, the surface sterilized roots were chopped into 1 mm sections, divided into 50 mg subsamples, and total DNA was extracted using PowerPlant DNA isolation kit (MoBio, Carlsbad, CA) with the following modifications relative to manufacturer's instruction. We added 50 ul of 10% cetyltrimethylammonium bromide to each lysis tube containing the lysis solution and roots to enhance plant cell lysis, followed by three freeze-thaw cycles (80°C/65°C; 10 min each) and homogenization in a mixer mill for 20 min at 30 Hz (model MM400; Retsch Inc., Newtown, PA). Three subsamples were then concentrated and combined into a single 50 ul extraction. PCR amplification of bacterial 16S rRNA gene from the genomic DNA of 92 (23 trees X 2 seasons X 2 endosphere/rhizosphere) samples was conducted using a pair of primer that targets the V6-V9 region of 16S. The fusion F1070F (5′-TCAGCTCGTGTYGTGARA-3′) and 1492R primers (5′- TACCTTGTTACGACTT-3′) were employed with modification for use with the GS FLX Titanium platform (454 Life Sciences, Branford, CT). These primers discriminate against plastid DNA and surrounded a ∼200 bp mitochondrial insert in *Populus*. Thus we excised and gel purified the bacterial enriched band prior to emulsion PCR. For each sample, the fusion forward primer was preceded by a unique 8 bp barcode, which was in turn preceded by the 454 A/B primers. For each sample, a 50 μl PCR reaction was conducted using the High Fidelity PCR system (Invitrogen, Carlsbad, CA), 0.2 mM of deoxyribonucleotide triphosphates (dNTPs), 2 mM MgSO_4_, and otherwise carried out as in Gottel et al. [15]. Fungal primers and conditions were identical to those used by Gottel et al. [15].

### Sequence Analysis

We denoised the pyrosequencing data using Ampliconnoise v1.27 [24], which corrects for both PCR and sequencing errors [25], through QIIME v1.4.0/1.5.0 [26]. Further, sequences that were less than 300 bp long and did not align with the SILVA database using MOTHuR v1.25 [27] were removed. The resulting high-quality sequences were then trimmed to 300 bp and binned to respective samples based on the unique barcodes. For bacterial samples, the sequences were then clustered using UCLUST [28] to representative OTUs at a sequence similarity of 97%. The representative sequences from OTUs were then checked for chimeras using ChimeraSlayer against the gold database provided with the software package. OTUs were assigned a taxonomic unit using RDP classifier 2.2 implementation of QIIME v1.4.0/1.5.0, and OTUs that were classified as chloroplast and archaea were removed from further analysis. A phylogeny of the representative sequence was built using the FastTree [29] algorithm in QIIME v1.4.0/1.5.0 after aligning with Pynast [30] algorithm against the GreenGenes [31] database. Further downstream analyses for weighted and unweighted Unifrac phylogenetic distance metric; Bray Curtis similarity metrics were all conducted in QIIME using a rarefied OTUs table to control for unequal sampling depths across samples. A principal coordinate analysis (PCoA) ordination based on the UniFrac distance matrix and the Bray Curtis metric was also generated. For fungal sequences, the sequences were checked for chimeras using implementation of UCHIME [32] in MOTHuR v1.25 without any reference sequences. UCHIME detects chimeras *de novo* with an assumption that chimeras are less abundant than their parent sequence. The sequences that were flagged as chimeras were then removed from further analysis. Any sequences that were less than 200 bp were also removed and the resultant sequences were then clustered into OTUs using UCLUST at sequence similarity of 97%. The representative sequences from OTUs were then assigned to taxonomic unit using RDP classifier 2.4 [33]. Raw sequence data and analysis files are available from the NCBI-BioProject data archive (http://www.ncbi.nlm.nih.gov/bioproject/?term=PRJNA209455) and our PMI project website (http://pmi.ornl.gov), respectively.

### Data Analysis

To investigate relationships between microbial community composition and environmental factors, tree genotype, seasonal variation and geographic distance between samples, we used vegan 2.0–5 (*capscale*) [34] for variance partitioning and ecodist [35] for partial mantel test in R statistical software [36]. The species by sample or OTU table that was used in the study was rarified to 1000 sequences per sample for bacteria and 400 for fungi. In order to conduct partial Mantel test with soil properties and tree properties listed in Table S1 and S2, we built a separate composite distance matrix from variables (after removing highly correlated ones) that were selected using the forward and backward selection against the corresponding UniFrac distance matrix. The matrix of composite variables was then generated based on Euclidean distance metrics using *dist* function in R. For genotype data, we generated a distance metrics based on SSR distance data using GGT2 [37]. For seasonal variation, which is a categorical data, we generated a distance matrix using the *daisy* command of R package *cluster*. We created geographical distance matrix between each tree using the location and compass direction (degree, minutes and seconds (DMS)) that were collected using a handheld GPS unit. The DMS format was converted to decimal degree (DD) using an online tool at http://transition.fcc.gov/mb/audio/bickel/DDDMMSS-decimal.html. The DD coordinates were then used to generate a geographic distance matrix by a platform free java based software Geographic Distance Matrix Generator. For variance partitioning, geographic and genotype distance matrices were converted to principal coordinates using PCNM (Principal Coordinates of Neighborhood Matrices) function in vegan [34] and only the significant coordinates were included in the model. The following normalizing transformations of the variables were done before performing multivariate analyses: Canopy, Basal Areas, River Distance, P, Ca, Mn, Zn, K, Mg, N, DBH, LBC (ppm CaCO_3_/pH) were log10 transformed; percentages of clay, silt, sand, C, OM, basal area dominance of *Populus spp*/hectare were arcsine transformed; count values of number of proximal trees (10x Prism-based) and number of proximal *Populus spp.* trees (10x Prism-based) were square root transformed, and pH values were left unchanged.

## Results

**Figure 1:**
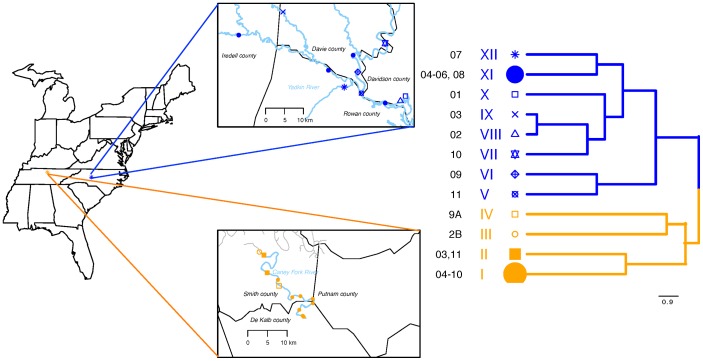
Map of sample locations along Caney Fork River in Tennessee and Yadkin River in North Carolina along with phylogenetic tree for the 23 individuals of *P. deltoides* generated from 20 simple sequences repeat markers. Each symbol on the maps represents the location of each tree sampled along the river and the corresponding symbol in the phylogenetic tree represents the genotypic relationships amongst individual hosts. The size of the symbol on the branch corresponds to the number of trees in each of the three putative host clonal groups.

We sampled roots [ca. 2 mm or less in dia.] from twenty-three *P. deltoides* individuals along watersheds of the Yadkin River (NC) and Caney Fork River (TN) over spring and fall seasons (May and September 2010) (Fig. 1). During the May sampling, we also collected bulk soil from three adjacent locations around each tree to characterize their physical and chemical properties. Geographic coordinates of the sites and the physical and silvicultural properties of host and surrounding environment were also assessed. For this study, host properties were comprised of measurements associated with host phenotype and surrounding silvicultural setting (including size and distance to nearest neighbor, distance to river, etc.). A comprehensive list of all the host and soil data that was recorded is listed in Tables S1 and S2.

Soils between the two watersheds differed significantly (p≤0.05) in numerous properties including Ca^2+^, CaCO_3_, K^+^, organic matter (OM), phosphate content and pH. A hierarchical cluster analysis of the measured host and environmental variables revealed high correlation between several of these measured factors. For instance, C and N (Spearman's ρ^2^ = 0.94), CaCO_3_ and OM (ρ^2^ = 0.89), % sand and % clay (ρ^2^ = 0.75), as well as silvicultural variables such as basal area and DBH (diameter at breast height) (ρ^2^ = 0.97) showed high correlation between pairwise combinations (Figs. S1 and S2). Thus, several of these highly correlated factors (Soil: N, CaCO_3_, % Sand and Host: DBH) were removed from downstream analysis to minimize redundancy within variance partitioning models employed.

Host genotype analyses based on twenty simple sequence repeat (SSR) primer pairs resulted in two distinct genetic groups, each comprised of individuals originating from either NC or TN with no overlap between the watersheds (Fig. 1). A total of forty-eight alleles were observed, with an average of 2.4 alleles per primer pair. A phylogenetic tree showing relationships between the individuals is also shown in Figure 1. The 20 microsatellites uniquely discriminated 11 of the 23 individuals evaluated. Fourteen individuals that could not be uniquely genotyped fell into three putatively clonal groups, two in TN populations and 1 in the NC population. Group I of TN was represented by eight individuals, group II in TN consisted of two individuals, and group XI was comprised of four individuals from NC. The overall geographic distance between trees including both NC and TN significantly correlated with pairwise genetic distance between the trees (Mantel test: ρ = 0.73, p = 0.0001) [35], [36]. However, within each local population these associations were much weaker and only the geographic distance between tree locations from the watershed in TN significantly correlated with genetic distance (ρ = 0.39, p = 0.02).

Barcoded 454 pyrosequencing of bacterial 16S rRNA and fungal 28S rRNA gene amplicons from 185 rhizosphere and endosphere samples resulted 946,354 high-quality reads after removing sequencing and PCR artifacts using AmpliconNoise [24] and ChimeraSlayer [38]. These sequences grouped into 24,435 bacterial OTUs (≥97% similarity) and 2,999 fungal OTUs. Table S3 and S4 summarize the sequencing reads and OTUs obtained for each rhizosphere and endosphere sample from bacteria and fungi along with number of OTUs. Unlike our previous efforts targeting the bacterial V4 region [15], the V6–V9 primer sets and gel separation procedures employed in this study were able to reduce the amount of host plastid and mitochondrial sequence coincidentally contained in bacterial endosphere samples to an average of ∼8%, from ∼85% on average in our previous study.

### Taxonomic distribution

**Figure 2:**
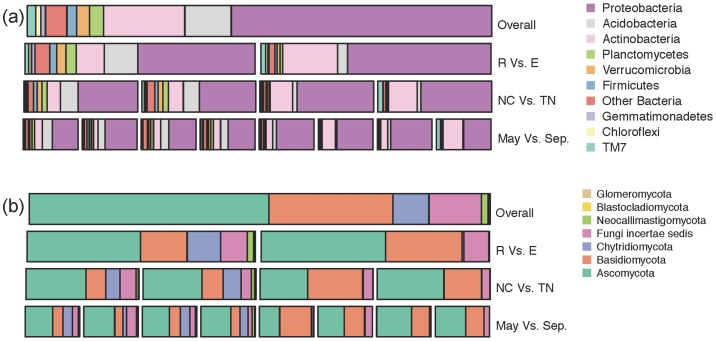
Taxonomic distribution of bacterial (A) and fungal (B) communities from roots of *Populus deltoides*. The first row of stacked bar represents the overall relative abundance across the entire data set, the second row represents endosphere vs. rhizosphere, third row represents the relative abundance in each watershed and the fourth row represents the relative abundance in May vs. September.

Across all samples, we detected a total of forty bacterial phyla from the rhizospheric and endospheric samples, but only nine had an average abundance greater than 1%. The phyla that made up most of *P. deltoides* overall root (rhizosphere and endosphere) microbiome were *Proteobacteria* (56.1%), *Actinobacteria* (17.5%), *Acidobacteria* (10.0%), *Firmicutes* (2.1%), *Planctomycetes* (3.0%), *Verrucomicrobia* (2.8%), *TM7* (1.8%), *Chloroflexi* (1.1%) *and Gemmatimonadetes* (1.0%) (Fig. 2A). Differential phyla level trends were observed in the rhizosphere and endosphere bacterial communities. In all rhizosphere samples, regardless of watershed or seasonal origin, *Proteobacteria* (51%) was the most abundant phylum followed by either *Actinobacteria* (12.1%) or *Acidobacteria* (14.6%). The remainder of the phyla showed high variability in abundance from sample to sample. For instance, relative abundance of *TM7* in a rhizosphere sample was as high as 19.1%, but its average abundance was only 1.6%. Whereas in the endosphere samples, *Proteobacteria* (62.4%), *Actinobacteria* (23.9%) were enriched, largely at the expense of *Acidobacteria* (4.3%), and members of the *Chloroflexi* (1.0%), *Planctomycetes* (1.1%), *TM7* (2%) and *Verrucomicrobia* (1.3%) were among the less abundant phyla. Endosphere samples exhibited much greater variability from sample to sample than those from the rhizosphere (Fig. S6). Also, unlike in rhizosphere samples, *Proteobacteria* were not always the most abundant phyla as in the endosphere, as *Actinobacteria* were dominant in ∼10% of samples.

We detected a total of eight fungal phyla in the rhizospheric and endospheric samples from *P. deltoides.* Across all samples, *Ascomycota* (52%) dominated the overall fungal communities – both rhizosphere and endosphere – followed by *Basidiomycota* (26.9%), *Chytridiomycota* (7.8%) and others of the largely unresolved basal lineages in the former *Zygomycota* (now *Mucorales*, *Mortierellales*, etc) that are reported here as *Fungi incertae sedis* (11.4%) (Fig. 2B). A similar trend was observed in the rhizosphere with *Ascomycota* (50%) as the most dominant phylum, followed by *Basidiomycota*, (20.5%), *Fungi incertae sedis* (11.7%) and *Chytridiomycota* (14.7%) (Fig. 2B). In contrast, overall endosphere communities consisted primarily of *Ascomycota* (55%), *Basidiomycota* (33%), and *Fungi incertae sedis* (11%), while *Chytridiomycota* were largely absent (<1%).

At the higher taxonomic levels we observed moderate differentiation over geographic space and season compared to differences between the rhizosphere and endosphere. For instance, seven out of the nine major bacterial phyla differed in abundance significantly between rhizosphere and endosphere (p≤0.05) (Fig. S3). Similarly, *Chytridiomycota* were completely absent from 50% of endosphere samples, and across all spatial and temporal samples, only reaching a total of 0.7% of endosphere sequences, yet was one of the dominant phyla in the rhizosphere samples. Other fungal phyla including *Basidiomycota, Blastocladiomycota* and *Neocallimastigomycota* also differed significantly between rhizosphere and endosphere communities (Fig. S4); however, only few phyla differed in their composition over space (i.e., watershed) and time (i.e., season) (Fig. S4). Compared to differences between rhizosphere and endosphere, we only observed moderate and often inconsistent differences within these communities, regardless their sampling location or season of collection (Figs. S3 and S4). Over space, *Chloroflexi* and *Ascomycota* from endosphere communities and *Blastocladiomycota*, *Acidobacteria* and *Chloroflexi* from rhizosphere communities were significantly different between trees from watersheds in NC and TN. Over the two seasons, *Glomeromycota* from endosphere of TN trees was the only fungal phyla that changed significantly from one season to another. In contrast, 10 bacterial phyla showed significant changes between the two seasons; however these seasonal patterns were often inconsistent between watersheds. For example, a significant shift between dominance of *Proteobacteria* (dominant in spring) and *Actinobacteria* (dominant in fall) in the root endosphere was observed between season in the TN samples, but not the NC samples. (Fig. S3).

### Factors related to microbial community patterns of the rhizosphere and endosphere

Pairwise UniFrac distances [39] between each sample indicated that bacterial and fungal communities from roots of *P. deltoides* varied significantly (p≤0.05) between rhizosphere and endosphere (p≤0.05, Fig. S3 and S5). Though the rhizosphere and endosphere from a common root sample were only millimeters apart, they displayed significant differences in major phyla (Fig. S3), number of OTUs (Tables S3 and S4), and UniFrac distance (Fig. S6). To further characterize these communities we separated rhizosphere and endosphere data in recognition that these likely represent separate habitats or niches that may have differing drivers of their community structure. To identify these drivers of microbial community structure we tested the relationships between community structure and various measurements that included host genotypes and phenotype, soil physical and chemical parameters, geographic distance between samples, season, the characteristics of the reciprocal community associates (bacterial vs. fungal) and the interactions of these variables. Using variance partitioning with distance-based redundancy analysis (db-RDA) of UniFrac inter-sample distances, we determined which host and environmental factors best explained the community structure [40], [41]. In our results, most of the variation in the community structure for both communities in rhizosphere and endosphere is statistically unexplained (>40%) with only few of the factors contributing significantly to the variance (∼20%, p≤0.05). Figure 4 represents the proportion of community variance explained by variation of individual factors (effects of all others are neutralized), interaction among factors, and the unexplained variance for both bacterial and fungal communities in rhizosphere and endosphere.

**Figure 3:**
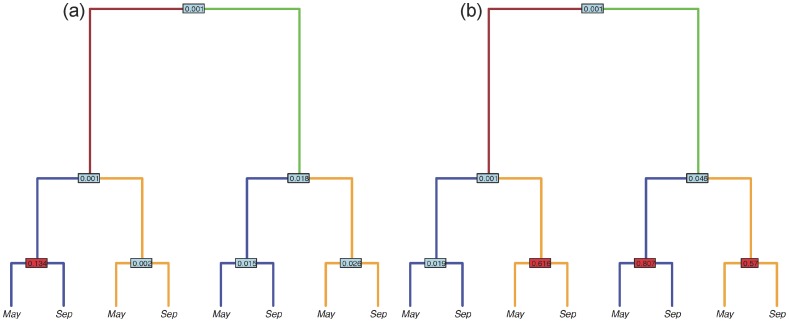
A phylogram-based illustration of the experimental design and difference in phylogenetic-based community structure between rhizosphere, endosphere, watersheds and seasons for bacterial (A) and fungal (B) communities. Rhizosphere is represented by brown edges and endosphere by green edges. Similarly, two the watersheds are represented by orange and blue edges for Tennessee and North Carolina, respectively. The end nodes represent the two seasons of sample collection. The number at the node represents the p-values (red for insignificant, blue for significant p≤0.05) generated by comparing the unweighted UniFrac distance metrics between two conditions (left and right nodes) using the *adonis* function of vegan package in R. Phylograms represent (A) bacterial and (B) fungal communities.

**Figure 4:**
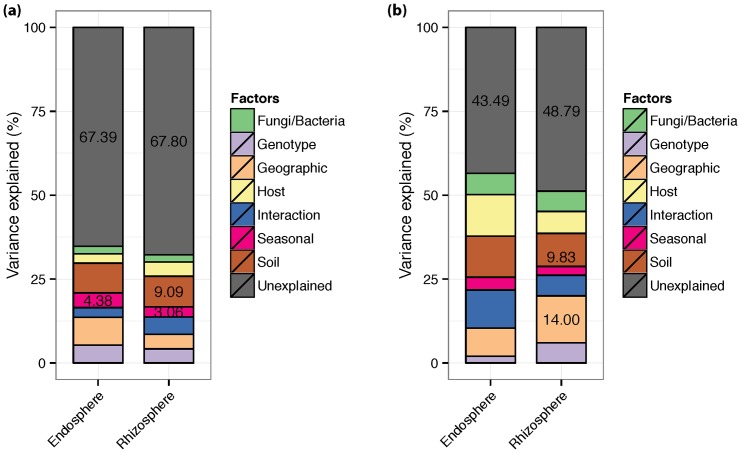
Variance partitioning of bacterial and fungal communities from the roots of *Populus deltoides* into soil properties, host properties, spatial, host genotype, seasonal and beta diversity of corresponding bacterial or fungal community. Each bar represents total variance partitioned into pure effect or interaction of two or all factors. Only variance proportions that were statistically significant (p≤0.05) are listed. Variables for host and soil properties were selected based on stepwise selection (forward and backward) to remove non-significant terms from the model. (A) Variance partitioning of bacterial community from endosphere and rhizosphere. (B) Variance partitioning of fungal community from endosphere and rhizosphere.

The most important factors that directly or indirectly affected the bacterial community in rhizosphere were soil properties and season. Based on the variance partitioning analysis, seasonal change (∼4%; p≤0.05) and soil properties (9.1%; p≤0.05) explained significant proportions of variances in pairwise UniFrac distances between samples (Fig. 4a). To elaborate on the component of soil properties, we plotted Canonical Correspondence Analysis (CCA) [42] with a subset of best factors – factors selected using stepwise selection – that made up the composite soil variable. Among all soil factors, pH had the largest effect on the bacterial rhizosphere communities (Fig. S7). Interestingly, differences in the rhizosphere bacterial communities are best explained by the variance in local soil properties like pH, despite significant differences between communities from two geographic populations (Fig. 3), indicating that the bacterial rhizosphere communities associated with *P. deltoides* are principally structured by changes in the local environment. In endosphere bacterial communities, only season significantly explained any variance (∼4%; p≤0.05), suggesting that both local environment and host properties have lesser influence on endosphere bacterial communities compared to changes due to season.

Unlike bacterial communities, significant proportions of variance in (14.0%; p≤0.05) UniFrac distance between fungal rhizosphere communities were explained by inter-tree geographic distances. Furthermore, soil properties explained 9.8% (p≤0.05) of variance in UniFrac distance between communities (Fig. 4b). A CCA plot of fungal communities from rhizosphere indicates a relationship between soil properties Ca, Mn and moisture content with fungal communities (Fig. S8). In contrast to bacterial rhizosphere communities, both the local environment and geographical settings, but not the host genotype, influenced fungal rhizosphere communities. For endosphere fungal communities, none of the factors that we measured explained significant proportion of variance.

### Correlation between fungal and bacterial communities

Over regional scales (both NC and TN watershed in unison), rhizosphere fungal and bacterial communities appear to have some reciprocal influences on each other. Here, partial Mantel tests [35] revealed that the UniFrac distances between fungal communities from rhizosphere are significantly correlated with UniFrac distances between bacterial communities (ρ = 0.24, p = 0.004) (Table S5). Similar to variance partitioning, these Mantel tests account for all other measured variables. However when we conducted the test separately on local NC and TN populations, significant correlations were only maintained among the rhizospheric populations in TN (ρ = 0.28, p = 0.03).

### OTUs distributions and the core microbiome

OTUs from roots can be divided into three categories based on their distribution: 1) rhizosphere-specific OTUs that are only found in rhizosphere, 2) shared OTUs that are found in rhizosphere and endosphere and 3) endosphere specific OTUs. Most of the OTUs (bacterial and fungal) in the roots were rhizosphere specific, with a few shared between the two habitats, and even fewer being endosphere specific (Table 1). However, while the number of unique rhizosphere OTUs is high, shared OTUs comprised of most of the sequences (82%), indicating greater dominance often enrichment in the endosphere compartment. Analysis of the distribution of the OTUs amongst each category showed that 77.8% of rhizosphere-specific OTUs and 90% of endosphere-specific OTUs were unique to one host sample and only few OTUs were present in all sampled trees. Similarly, there were shared OTUs that were only found in one host, but most shared OTUs were present in multiple host samples. For instance, among shared OTUs, 85% and 53% were found in multiple tree rhizosphere and endosphere samples, respectively. We also detected shared OTUs that were present in rhizosphere or endosphere samples of all trees. A set of 34 OTUs that were shared and one rhizosphere-specific OTU constituted putative ‘core’ rhizosphere microbiome of *P. deltoides* from the two populations. One of the core OTUs from the rhizosphere was also detected in endosphere of every sampled tree. A table with the list of core OTUs along with their top BLAST hits against reference genomic sequences database in NCBI website is listed in Table S6. These core OTUs in rhizosphere mostly consist of *Proteobacteria* and at lower frequencies *Actinobacteria, Acidobacteria, Verrucomicrobia* and *Chloroflexi*.

**Table 1:** Distribution of rhizosphere specific, shared, and endosphere specific bacterial and fungal OTUs among all the sampled trees.

	Bacteria				Fungi			
# of Trees	Rhizosphere specific	Shared (Rhizosphere)	Endosphere specific	Shared (Endosphere)	Rhizosphere specific	Shared (Rhizosphere)	Endosphere specific	Shared (Endosphere)
1	4920	127	328	410	769	72	101	126
2	683	102	24	178	174	30	11	35
3	255	84	9	84	59	21	3	23
4	152	64	3	50	31	17	3	10
5	88	53	2	27	19	21	2	9
6	60	45	0	18	11	10	3	9
7	37	41	0	22	10	14	1	4
8	28	33	1	8	6	6	0	12
9	19	30	1	10	5	11	0	5
10	20	32	0	6	2	8	0	5
11	13	19	0	5	3	5	0	3
12	11	16	0	6	1	3	0	2
13	11	11	0	3	1	5	0	3
14	2	17	0	3	0	3	0	3
15	5	18	0	3	0	3	0	0
16	4	17	0	4	0	5	0	1
17	1	21	0	2	0	3	0	0
18	4	17	0	3	0	4	0	1
19	2	13	0	8	0	3	0	1
20	2	18	0	6	0	2	0	1
21	0	22	0	1	0	3	0	0
22	1	23	NA	NA	0	1	0	0
23	1	34	NA	NA	0	4	0	1

Two endosphere samples that repeatedly failed to amplify are listed as NA in the table.

We observed similar distribution of fungal OTUs between rhizosphere and endosphere and among trees. Here 70% of rhizosphere-specific and 81% of endosphere-specific OTUs were only detected in only one host sample (Table 1). We also found shared fungal OTUs that were specific to a single tree, but most were found in multiple trees, as 71% of shared OTUs in rhizosphere and 50% of endosphere were common to multiple trees. The shared OTUs constitute a putative ‘core’ set of rhizosphere OTUs that were comprised of 4 OTUs. One of the four OTUs was found in endosphere of all the sampled trees. Three out of four core OTUs from rhizosphere were classified as *Ascomycota* and the other as a *Mortierella* sp. (Table S7).

## Discussion

### Differences in rhizosphere and endosphere communities

We previously conducted a study of two locations near the Caney Fork River in TN that revealed that the rhizosphere and endosphere communities of *P. deltoides* were distinct across both their bacterial and fungal communities [15]. With the current study we show this pattern clearly holds across a much wider range of soil types, seasonal transitions, host characteristics and across two regions in the southeastern USA. Additionally, the present study further delineates the phyla and OTUs that are contributing to these differences between rhizosphere and endosphere communities and recovers a greater range of microbes than was revealed in the previous study. At higher taxonomic levels, we observed *Acidobacteria* and *Chytridiomycota* were both more abundant in rhizosphere compared to the endosphere. This result is consistent with recent results reported for studies of the roots of *Arabidopsis*, which also reported low levels of *Acidobacteria* in the endosphere [20]. These two reports suggest that members within *Chytridiomycota* and *Acidobacteria* phyla may lack properties essential for proliferation within endophytic environments.

In contrast to our previous work, we found *Actinobacteria*, similar to the genus *Streptomyces*, were sometimes as dominant (or more so) within endophytic samples as *Pseudomonas*-like *Gamma-proteobacteria*. Our recent study using ‘synthetic community’ mixtures of known composition have shown that the V4 primer set we used in our previous study underrepresented *Actinobacteria* in community analyses [43]. In the current study we employed new primers targeted at the V6-9 region and additional methods to reduce host plastid and mitochondrial rRNA gene contamination. The primer set was tested against this ‘synthetic community’, which revealed that V6-9 set was able to better recover the overall bacterial diversity, including *Actinobacteria* (Fig. S9) [43]. Beyond reducing plant organelle sequence (averaging ∼85% in our endosphere samples in the previous study to ∼8% in the present), these methods also appear to have eliminated previous biases against *Actinobacteria* taxa. This prominence of *Actinobacteria* in the endosphere is also consistent with other recent studies of plant root endophytes [20]. Detailed follow-up studies with isolates of these phyla may provide valuable insights into deciphering genotypic and phenotypic properties of hosts and microbes that contribute to the entry, survival, growth and function within host habitats.

### Factors governing rhizosphere and endosphere community composition

Our analyses employed variance-partitioning methods to understand how combinatorial effects of host factors, soil properties, presence of other microbes and seasonal variation effect plant associated microbial communities. However, a quantitative understanding of the relative importance of each of factors remained elusive in our study, especially for endosphere communities, that exhibited low diversity (average OTUs: 154 (Bacteria), 39 (Fungi)), but high variability from sample to sample (ranging from 19–1079: bacterial OTUs and 8–169: fungal OTUs). Given the large amounts of unexplained variance within our study, despite considerable efforts to measure a diverse suite of host and environment associated variables; it is possible that unmeasured and/or stochastic factors may play large roles in formation of endosphere and rhizosphere communities. However, given the diverse nature of these communities compared to the relatively low sample sizes we employed (derived from 23 trees, tracked over two seasons in two watersheds), it is quite likely that more of this variation may be attributable with a more robust sample size. Additionally, our power to observe differences given these sample numbers was also likely limited by the significant amount of co-variation that occurred across the two watersheds/populations we sampled, as a variety of soil properties and host genotype differed significantly between the TN and NC sample origin.

Despite these limitations several factors were found to have significant effects on the structure of rhizosphere and endosphere communities. Within rhizosphere communities the effects of several soil properties (especially pH), while not large, were significant across both seasons and regions for both bacterial and fungal communities (Fig. 4 and S5). Such results have also been observed in previous studies [44]–[46]. Season of sampling also consistently explained a significant proportion of variance in bacterial communities of the rhizosphere, however was not consistently significant in explaining variation within fungal communities (Fig. 3). Additionally, both bacterial and fungal community properties varied strongly within the region in which they were sampled (TN vs. NC). The overall observations agree well with previous studies that have identified soil type and season as important players in shaping the microbial community of plants [16], [17], [47]. The importance of geographic distances in structuring fungal communities may also be due to greater dispersal limitations of fungi than for bacteria, leading to larger effects due to isolation by distance [48].

Both bacterial and fungal community structure within rhizosphere were shown to have influences upon each other in the TN population samples (e.g., bacterial community structure correlated with fungal community structure and vice versa). Such interactions, especially bacterial community structures being dependent on corresponding fungal diversity have been documented in other cases [49]–[51]. Bacterial influence on fungi, while well documented within studies conducted on Petri plates, are less well documented in natural systems [52]. The correlations may be indicative of relationship across these groups through the production of secondary metabolites, anti-microbial compounds and/or physical contact [53]. For instance, enzymatic activity of extracellular fungal enzymes in lignocellulose-rich soil environments that results in production of water-soluble sugars and phenolic compounds serve as growth substrates for bacteria [54].

Plant genotypic effects on microbial community in and around the roots have been documented in other host species [19], [55]. Based on the twenty SSR markers that we employed across both natural populations in our study, these influences were not significant. However, there was a large degree of covariance in our data sets, such that genetic relatedness measured with the SSR markers, as well as multiple soil properties, tended to co-vary between the two regional sampling areas. So while regional distinctions in both rhizosphere and endosphere microbiomes were clearly evident in our data sets (Fig. 3a and 3b) the specific influence of host versus environmental drivers on these differences remain mostly unexplained, and at least in part likely due to this high degree of covariance between variables across the two regions sampled. However, even within the three putative clonal types (ramet genotypes) identified in our SSR analysis, variation was not significantly different than between genotypes as measured by UniFrac distances (Fig. S6). Host influences on the microbial assemblages in the rhizosphere are complex, but may occur through factors affecting soil properties such as the release of rhizodeposits and exudates [56]–[58], secondary metabolites and other factors that were beyond the scope of variables tracked in this study.

### The ‘core’ endosphere and rhizosphere microbiome of Populus deltoides

A core microbiome is defined as members of the community that are found in all of the assemblages associated with a habitat [59], [60]. Deciphering the core microbiome has been proposed to be fundamental in understanding the ecology of a microbial community, as the groups of species that are commonly occurring in all habitats are likely to play important role towards communities' function [60]. We defined the core endosphere and rhizosphere microbial OTUs associated with all the sampled trees in the study (regardless of season, genotype, regional location, etc.) using rarified data sets that may exclude some common, but low abundance organisms compared to using overall (unrarified) distributions. These conservative approaches resulted in a rather narrow core microbiome in each habitat. Our core bacterial microbiome in rhizosphere was comprised of only 35 OTUs, one of which, a *Methylibium*-like OTU within *Burkholderiales*, was also the only member of the core endosphere microbiome (Table S6). Most of these core rhizosphere OTUs were within the order of *Burkholderiales* and *Rhizobiales* which are known to be important plant associated organisms, as well as to contain diverse gene clusters encoding degradation pathways for an array of aromatic compounds including pollutants [61].

The core fungal microbiome constituted only four rhizosphere OTUs and one endosphere OTU. Sequence analysis of fungal core OTUs in rhizosphere and endosphere revealed members likely represented the genera *Exophiala, Metarhizium, Neonectria sp.* and *Mortierella.* Some of these organisms are known to have positive benefits to the plants by increasing plant growth, preventing oxidative damage, mitigating salt stress, transferring nitrogen from insect to plant and/or acting as entomopathogens [62], [63]. *Neonectria* is known as an opportunistic plant pathogen in some environments; however their function within native rhizosphere habitats of *P. deltoides* remains undefined. Further genome sequencing of isolates, controlled inoculations and other experiments to test the molecular basis of these associations with host plants will be required to fully appreciate the roles and functions of these fungi.

## Conclusion

Analysis of rRNA gene amplicon pyrosequencing data from 23 *P. deltoides* host trees across two watersheds and over two seasons for fungal and bacterial communities revealed new details about the microbes and microbial community structure in the roots of *P. deltoides.* At higher taxonomic levels (e.g., phyla) rhizosphere and endosphere communities were highly similar between two watersheds differing only in abundance of major phyla. However, at finer levels such as methods using OTUs or UniFrac distances that account for overall phylogenetic variation, clear distinctions were observed for communities from different watersheds suggesting that mature plants of the same species in different locations harbor distinct microbial communities in and on their roots. Also, we observed a seasonally dynamic bacterial community in both the rhizosphere and endosphere of *Populus*. The high degree of covariation within the host and environmental datasets likely limited the power to distinguish between many of the genotypic, geographic and environmental factors that may shape the *Populus* microbiome. Future studies with more extensive sampling and in-depth host characterization should further elucidate the factors shaping community structure of both rhizosphere and endosphere communities in *Populus*. Fungal and bacterial OTU distribution across samples suggested a small set of OTUs that formed the core microbiome and should guide isolate studies that target the detailed mechanisms of host-microbe interactions in *Populus*.

## Supporting Information

Figures S1 Cluster analysis of the measured environmental variables (transformed) to remove redundant variables from the model. The analysis was done using varclus function of *Hmisc* package in R statistical software. (**S1**): Tree and stand properties (See Table S1 for data) (**S2**): Soil properties.
(PDF)

Figure S2 Cluster analysis of the measured environmental variables (transformed) to remove redundant variables from the model. The analysis was done using varclus function of *Hmisc* package in R statistical software. (**S1**): Tree and stand properties (See Table S1 for data) (**S2**): Soil properties.
(PDF)

Figures S3 Comparative analysis of major phyla between rhizosphere and endosphere, the two watershed populations and seasons for bacteria (S3) and fungi (S4). The significant difference is calculated using t-test between relative abundance of each pairwise phylum. Each bar represents relative abundance; the red labels represent significant differences (p≤0.05).
(PDF)

Figure S4 Comparative analysis of major phyla between rhizosphere and endosphere, the two watershed populations and seasons for bacteria (S3) and fungi (S4). The significant difference is calculated using t-test between relative abundance of each pairwise phylum. Each bar represents relative abundance; the red labels represent significant differences (p≤0.05).
(PDF)

Figure S5 Principle coordinate analysis of Unweighted UniFrac distance for bacterial (left) and fungal (right) communities. The plot indicates the rhizosphere and endosphere communities are distinct for both bacteria and fungi. Average Unweighted UniFrac distance matrix was calculated from 999 even rarefactions of 1000 sequences per sample for bacteria and 400 sequences per sample for fungi. Significance was calculated using *adonis* function of *vegan* package in R.
(PDF)

Figure S6 Boxplot of UniFrac distances comparing between and within host environments, genotypes, geographic populations and seasons.
(PDF)

Figures S7 Canonical correspondence analysis (CCA) of OTUs and soil and host factors on community composition for bacteria (S7) and fungi (S8). Larger circles represent samples that are color-coded based on their location; smaller dots represent species/OTUs.
(PDF)

Figure S8 Canonical correspondence analysis (CCA) of OTUs and soil and host factors on community composition for bacteria (S7) and fungi (S8). Larger circles represent samples that are color-coded based on their location; smaller dots represent species/OTUs.
(PDF)

Figure S9 A representation of accuracy of V6–V9 primers and methods against a synthetic community described in our previous study [43].
(PDF)

Table S1 Table of Tree locations, host and site/stand properties.
(XLSX)

Table S2 Table of soil physical and chemical properties averaged from 3 replicate bulk soil collections taken surrounding each tree/site.
(XLSX)

Table S3 Table of number of bacterial sequences, OTUs, and Chao index values for all samples. Sample name is abbreviation of its niche, season, location, and tree identity (R: Rhizosphere, E: Endosphere, M: May, S: September, N: North Carolina, T: Tennessee). For example R.M.N.01 represents Rhizosphere sample, collected in May from North Carolina tree #1. Similarly, E.M.N.01 represents Endosphere sample, collected in May from North Carolina tree #1.
(XLSX)

Table S4 Table of number of fungal sequences, OTUs, and Chao index values for all samples. Sample name is abbreviation of its niche, season, location, and tree identity (R: Rhizosphere, E: Endosphere, M: May, S: September, N: North Carolina, T: Tennessee). Also see legend of Table S3.
(XLSX)

Table S5 Results of mantel tests and partial mantel tests for key variables and UniFrac community matrices.
(XLSX)

Table S6 Detailed list and closely related organisms for each core bacterial OTU.
(XLSX)

Table S7 Detailed list and closely related organisms for each core fungal OTU.
(XLSX)
