# Supplementary figures and images for: A Multifactor Analysis of Fungal and Bacterial Community Structure in the Root Microbiome of Mature Populus deltoides Trees
Source: PLoS One. 2013 Oct 16;8(10):e76382. doi: 10.1371/journal.pone.0076382 (PMC3797799; doi:10.1371/journal.pone.0076382)

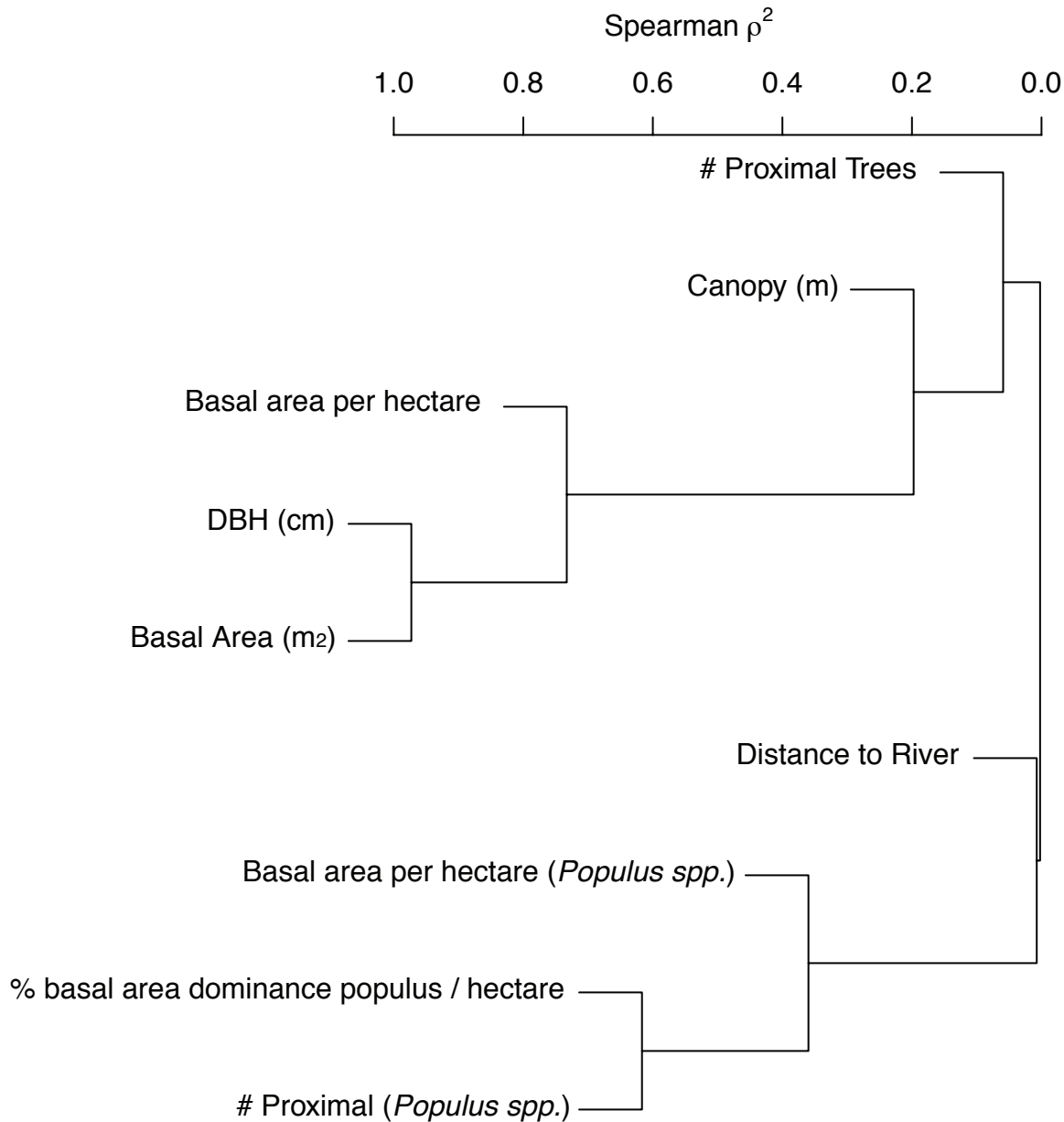

Supplement: Figures S1 — Cluster analysis of the measured environmental variables (transformed) to remove redundant variables from the model. The analysis was done using varclus function of Hmisc package in R statistical software. (S1): Tree and stand properties (See Table S1 for data) (S2): Soil properties. (PDF) [file pone.0076382.s001.pdf]

Spearman  $\rho^2$

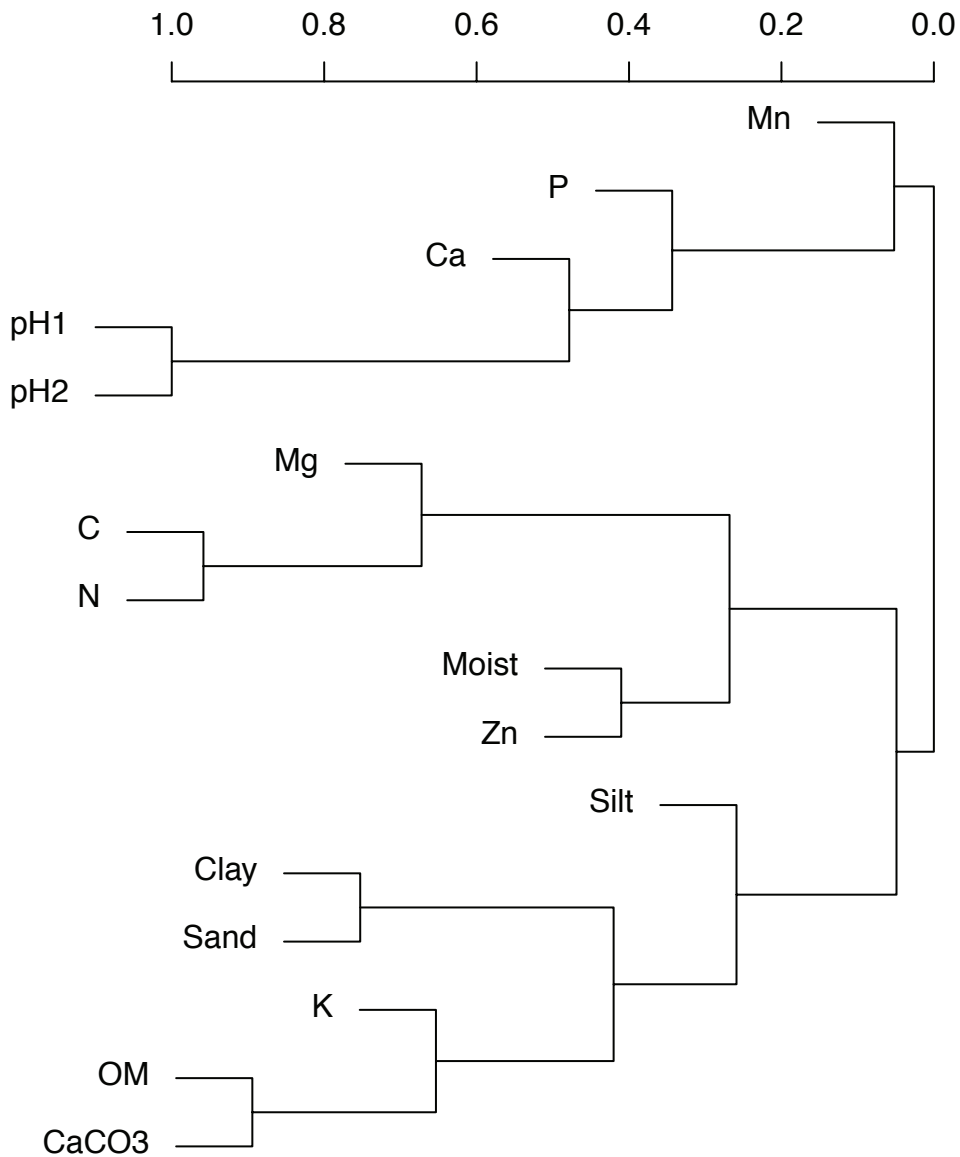

Supplement: Figure S2 — Cluster analysis of the measured environmental variables (transformed) to remove redundant variables from the model. The analysis was done using varclus function of Hmisc package in R statistical software. (S1): Tree and stand properties (See Table S1 for data) (S2): Soil properties. (PDF) [file pone.0076382.s002.pdf]

**Unweighted Unifrac Bacteria**

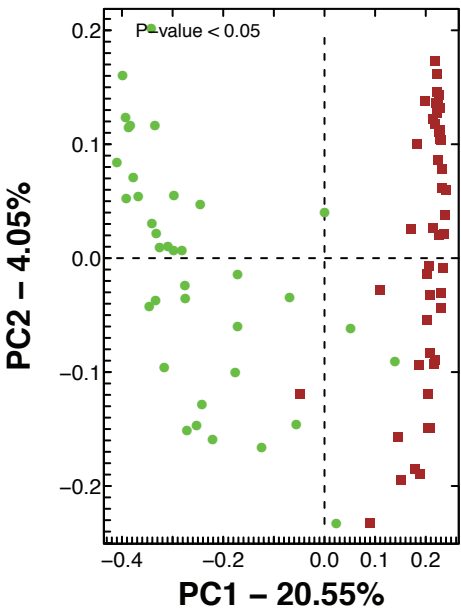

**Unweighted Unifrac Fungi**

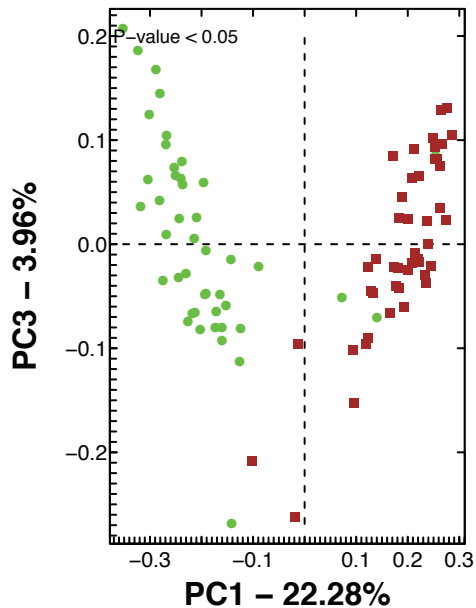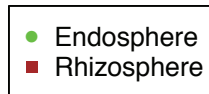

Supplement: Figure S5 — Principle coordinate analysis of Unweighted UniFrac distance for bacterial (left) and fungal (right) communities. The plot indicates the rhizosphere and endosphere communities are distinct for both bacteria and fungi. Average Unweighted UniFrac distance matrix was calculated from 999 even rarefactions of 1000 sequences per sample for bacteria and 400 sequences per sample for fungi. Significance was calculated using adonis function of vegan package in R. (PDF) [file pone.0076382.s005.pdf]

## Bacteria

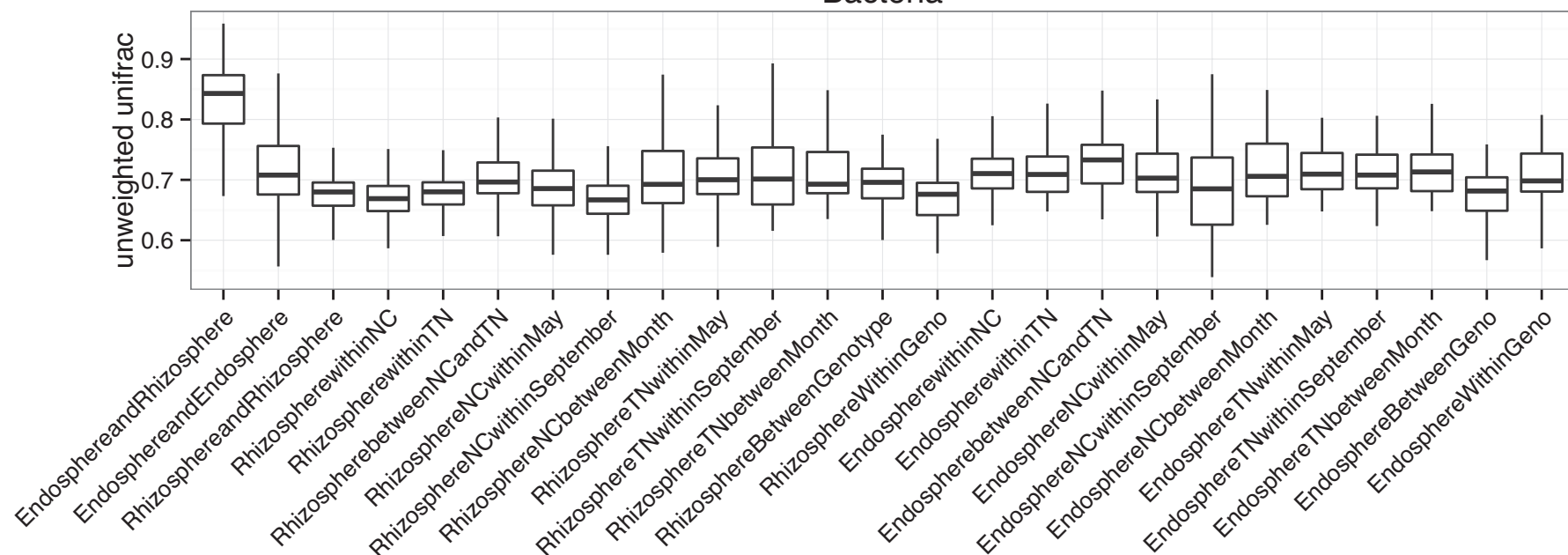

## Fungi

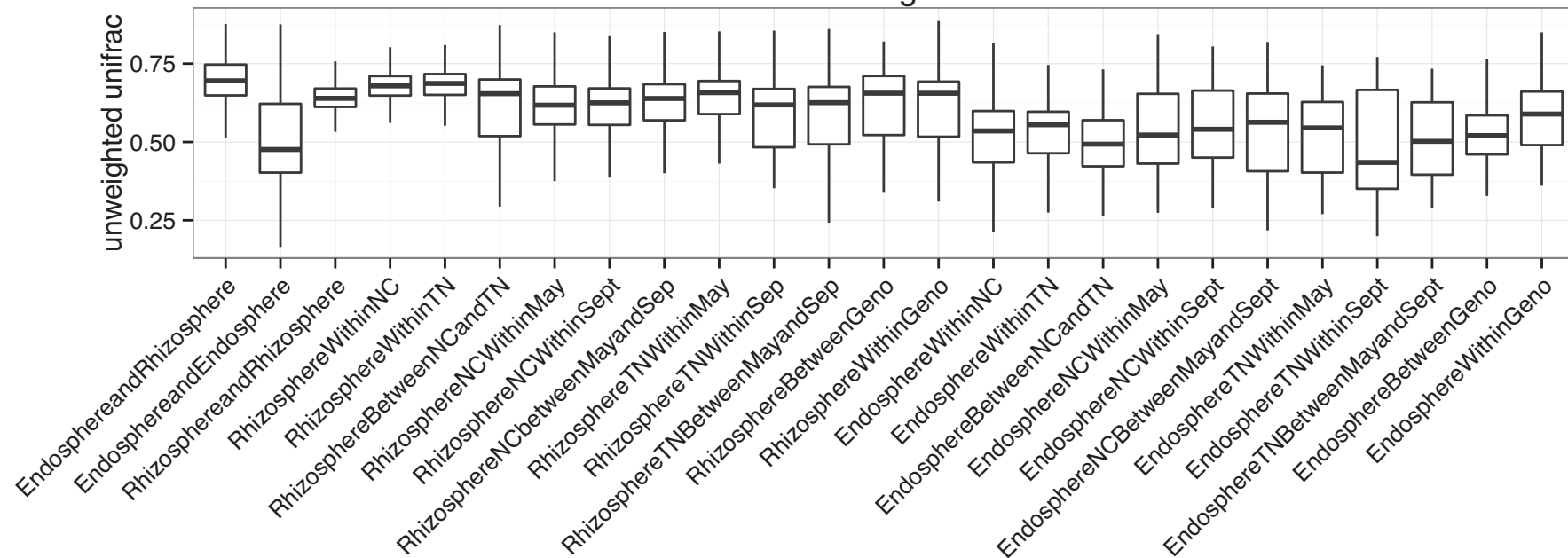

Supplement: Figure S6 — Boxplot of UniFrac distances comparing between and within host environments, genotypes, geographic populations and seasons. (PDF) [file pone.0076382.s006.pdf]

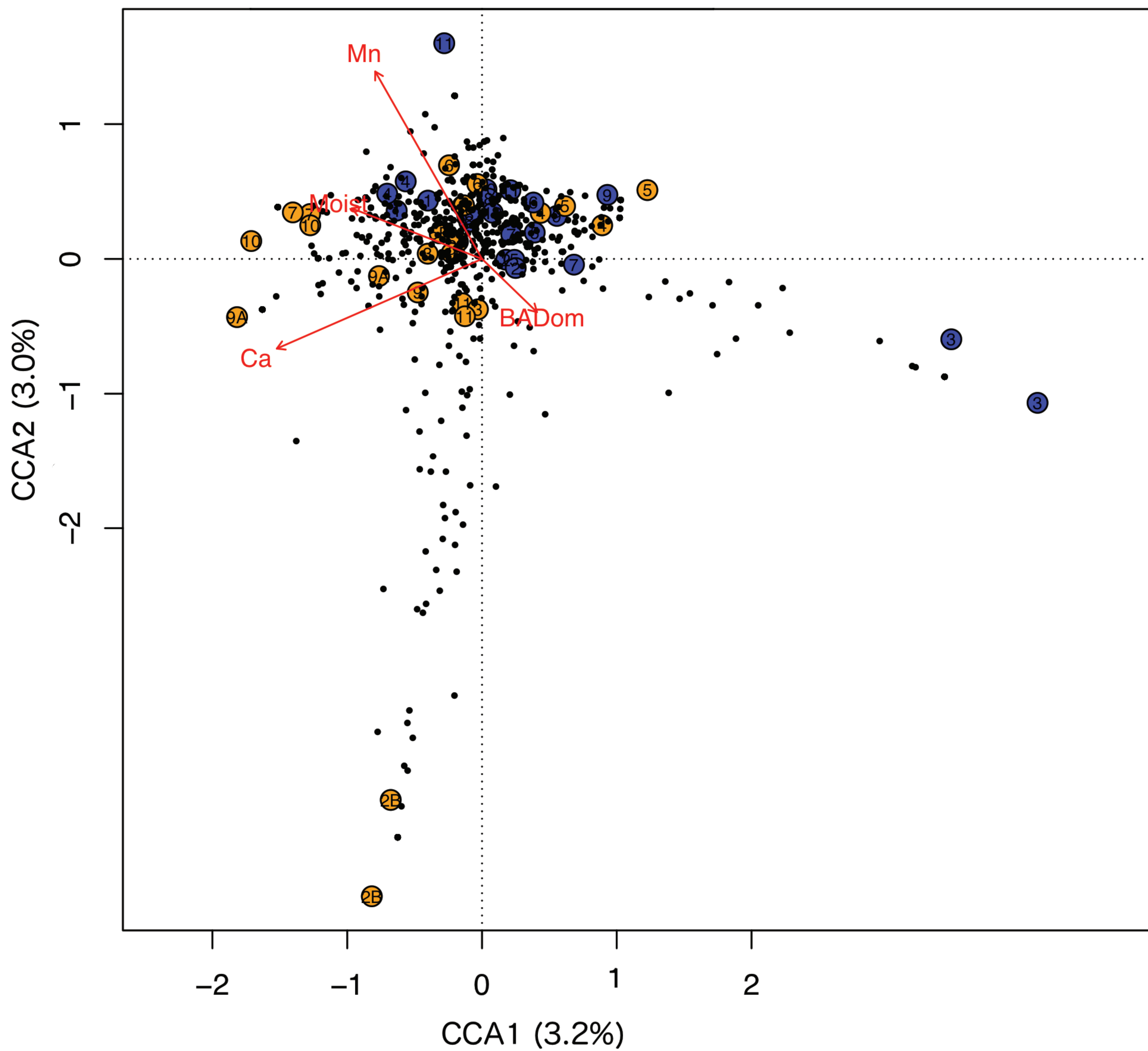

Supplement: Figure S8 — Canonical correspondence analysis (CCA) of OTUs and soil and host factors on community composition for bacteria (S7) and fungi (S8). Larger circles represent samples that are color-coded based on their location; smaller dots represent species/OTUs. (PDF) [file pone.0076382.s008.pdf]

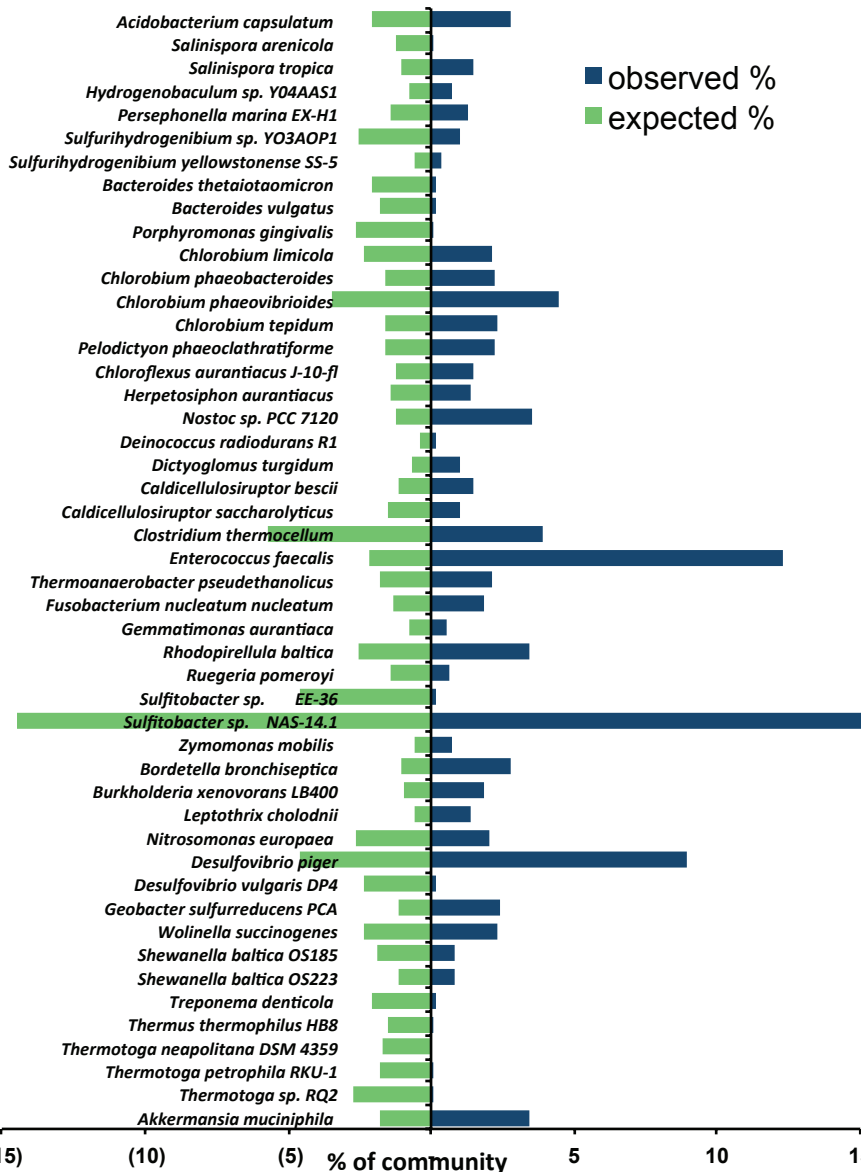

Supplement: Figure S9 — A representation of accuracy of V6–V9 primers and methods against a synthetic community described in our previous study [43]. (PDF) [file pone.0076382.s009.pdf]
