# Supplementary material for: A Multifactor Analysis of Fungal and Bacterial Community Structure in the Root Microbiome of Mature Populus deltoides Trees
Source: PLoS One. 2013 Oct 16;8(10):e76382. doi: 10.1371/journal.pone.0076382 (PMC3797799; doi:10.1371/journal.pone.0076382)
Supplement: Figure S4 — Comparative analysis of major phyla between rhizosphere and endosphere, the two watershed populations and seasons for bacteria (S3) and fungi (S4). The significant difference is calculated using t-test between relative abundance of each pairwise phylum. Each bar represents relative abundance; the red labels represent significant differences (p≤0.05). (PDF) [file pone.0076382.s004.pdf]

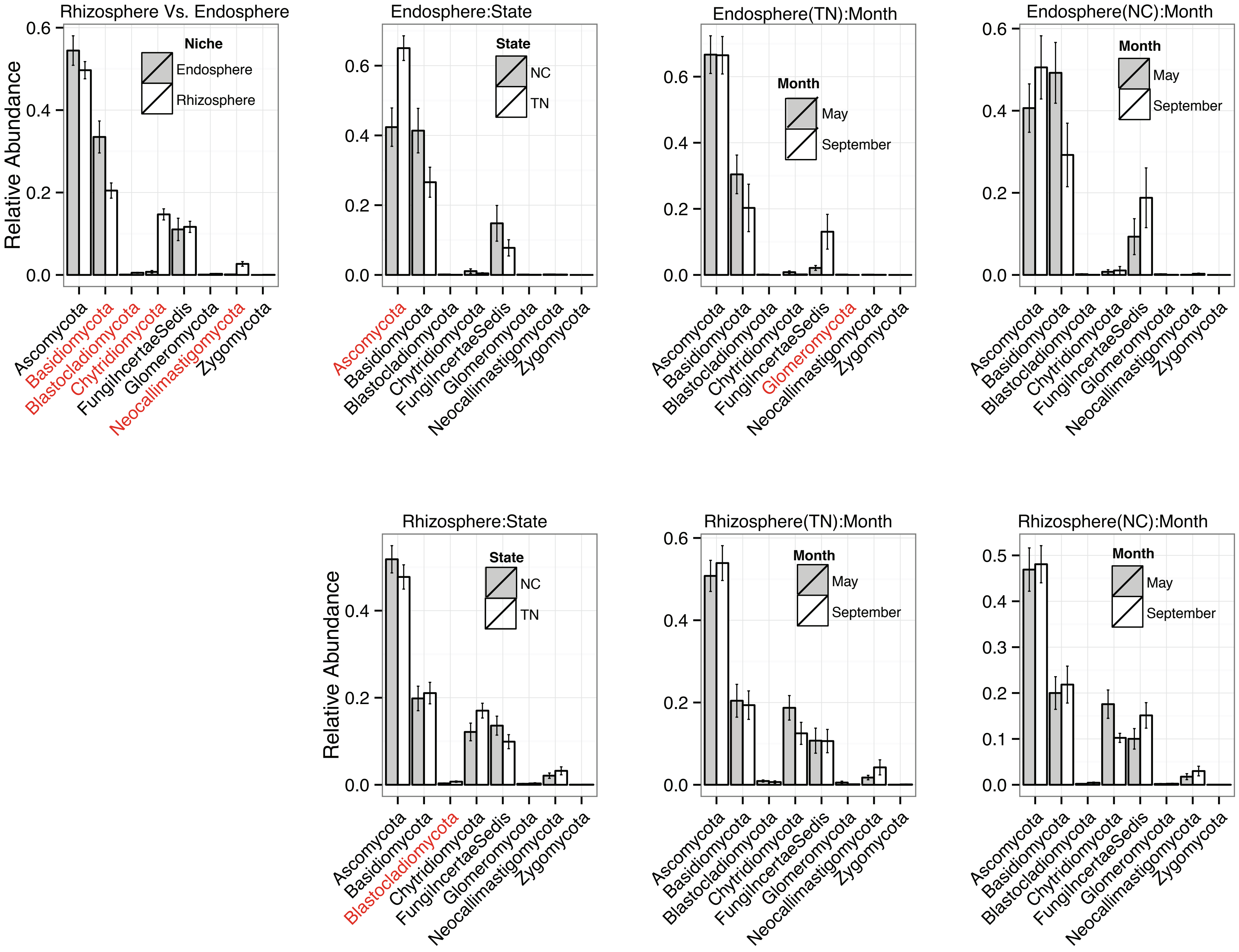

Rhizosphere Vs. Endosphere
Niche
Endosphere
Rhizosphere
Relative Abundance
Endosphere:State
State
NC
TN
Endosphere(TN):Month
Month
May
September
Endosphere(NC):Month
Rhizosphere:State
Rhizosphere(TN):Month
Rhizosphere(NC):Month
Ascomycota
Basidiomycota
Blastocladiomycota
Chytridiomycota
FungiIncertaeSedis
Glomeromycota
Neocallimastigomycota
Zygomycota
