# Supplementary material for: A Multifactor Analysis of Fungal and Bacterial Community Structure in the Root Microbiome of Mature Populus deltoides Trees
Source: PLoS One. 2013 Oct 16;8(10):e76382. doi: 10.1371/journal.pone.0076382 (PMC3797799; doi:10.1371/journal.pone.0076382)
Supplement: Figures S7 — Canonical correspondence analysis (CCA) of OTUs and soil and host factors on community composition for bacteria (S7) and fungi (S8). Larger circles represent samples that are color-coded based on their location; smaller dots represent species/OTUs. (PDF) [file pone.0076382.s007.pdf]

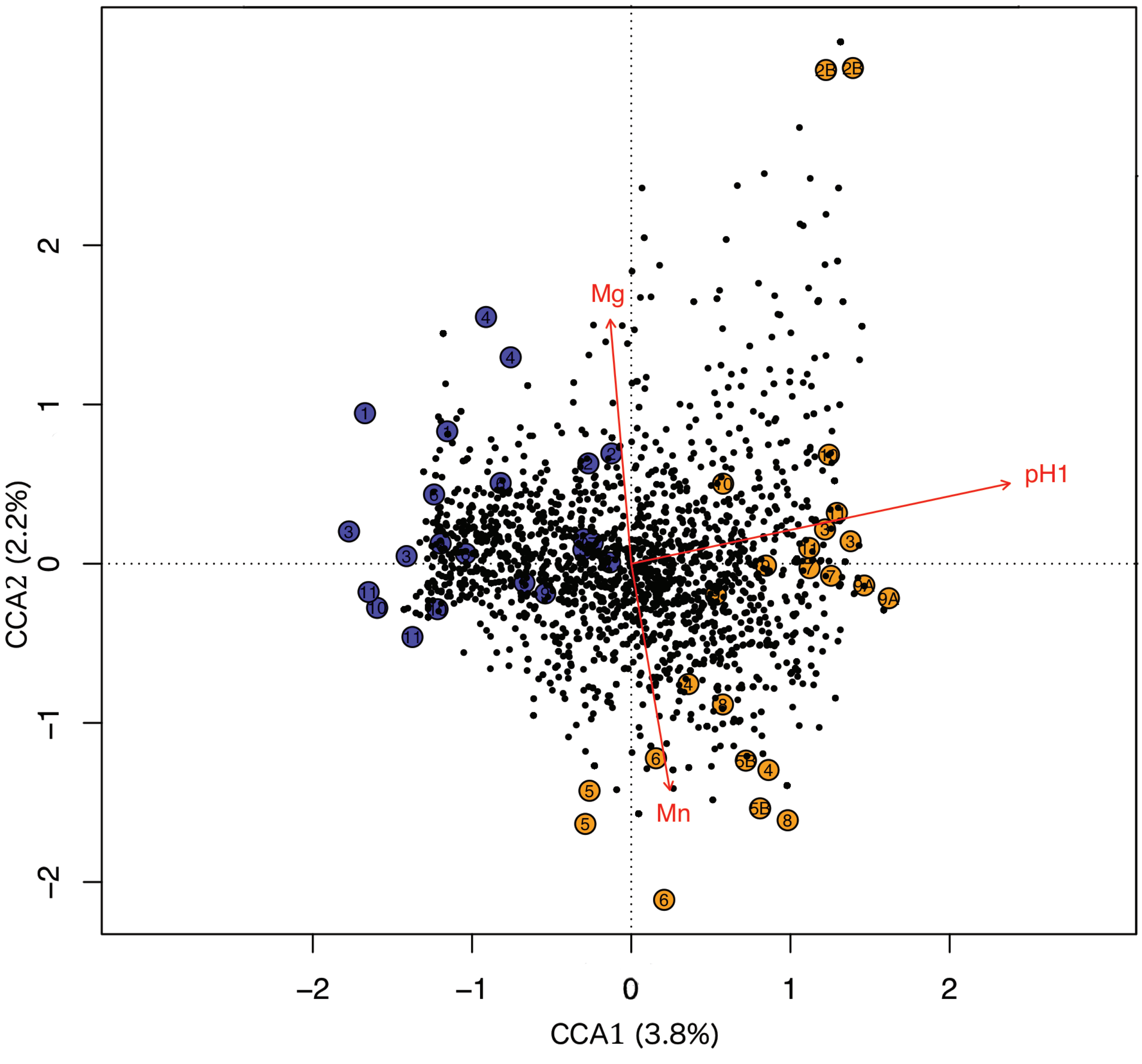

Mg
pH1
Mn
CCA2 (2.2%)
CCA1 (3.8%)
−2
−1
0
1
2
